# Cardiac Arrest and Cardiopulmonary Resuscitation: Starting from Basic Science and Bioengineering Research to Improve Resuscitation Outcome

**DOI:** 10.1155/2014/737542

**Published:** 2014-12-31

**Authors:** Giuseppe Ristagno, Tommaso Pellis, Yongqin Li

**Affiliations:** ^1^Department of Cardiovascular Research, IRCCS-Institute for Pharmacological Research “Mario Negri”, Via La Masa 19, 20156 Milan, Italy; ^2^Department of Anesthesia and Intensive Care, University Affiliated Hospital “Santa Maria degli Angeli”, Via Montereale 24, 33170 Pordenone, Italy; ^3^School of Biomedical Engineering, Third Military Medical University and Chongqing University, 30 Gaotanyan Main Street, Chongqing 400038, China

Cardiovascular disease remains the leading cause of death in the Western world with as many as 350,000 Americans and 700,000 Europeans sustaining cardiac arrest each year. Despite major efforts to standardize cardiopulmonary resuscitation (CPR) interventions, average survival rate presents a large variation all over the world [1–3]. Moreover, besides the initial success of CPR, the majority of victims die within 72 hours, due to the postcardiac arrest syndrome [4].

This difference in successful outcome may be mainly related to the overall diversity and strength in local organizations and algorithms in pre- and postresuscitation care. Indeed, resuscitation is a relatively modern science, although its roots extend back in the centuries. Yet, as early as the 19th century, resuscitation by delivery of an electrical shock was demonstrated. Modern CPR, however, emerged only during the latter half of the 20th century, with the sequence of interventions established in the 1960s under the acronym ABCD: airway, breathing, chest compression, and defibrillation [5]. Since then, novel therapeutic approaches have been conceived, introduced, and tested as new knowledge and pathophysiology understanding of cardiac arrest increased. Nevertheless, due to the complexity and interplay of events occurring during cardiac arrest and after resuscitation, events and mechanisms involved in resuscitation outcome are not completely understood [6]. This special issue on cardiac arrest and CPR, therefore, introduces brilliant contributions from worldwide experts in the field of resuscitation, arising and stimulating new strategies to improve outcome.

More specifically, space was provided to basic research on pathophysiology of cardiac arrest as well as to bioengineering developments. Thus, more clinically relevant and severe models of cardiac arrest, that is, with an underlying acute myocardial ischemia, a condition present in more than 70% of cardiac arrest events, are presented, together with investigations on the role of progressive mitochondrial ischemia during cardiac arrest and a focus on the quality of chest compression as determinant of resuscitation [7, 8]. Bioengineering research is also presented with the introduction of new computerized approaches to ameliorate CPR and postresuscitation care, including an reliable automated cardiac rhythm analysis during chest compression, in order to reduce detrimental interruptions in CPR [9]; an innovative quantitative characterization of early postresuscitation electroencephalogram; and an efficient automatic analysis of data, documentation, and information recorded during resuscitation. Finally, the quality of education and training programs on CPR are other critical factors in improving the effectiveness of resuscitation [10]. Survival rates after cardiac arrest depend, in fact, not only on the validity and on reliability of guidelines and a well-functioning chain of survival, but also on the quality of education. Thus, a report on a different teaching approach engaging trainees in the assessment of peer performance has been presented.

Translational research is a continuum loop in which basic science discovering is integrated into clinical application, while clinical observations are used to generate scientific topics to be studied by basic science. This integration is extremely important for medicine improvement [11]. Indeed, advances in resuscitation science have improved resuscitation care and ultimately survival of cardiac arrest over the years. The present issue aimed to improve such knowledge although only a limited number of researchers have found the appropriate space. Thus, special journal issues providing visibility to new upcoming idea and hypothesis in resuscitation basic science, translational studies, and bioengineering, like the present issue, have to be supported, paving the way towards a better comprehension of pathophysiology, mechanisms, and management of sudden cardiac arrest and amelioration of resuscitation care.

*Giuseppe  Ristagno* *Tommaso  Pellis* *Yongqin  Li*
